# Butyrate attenuates sympathetic activation in rats with chronic heart failure by inhibiting microglial inflammation in the paraventricular nucleus: Butyrate and sympathetic activation in chronic heart failure

**DOI:** 10.3724/abbs.2024092

**Published:** 2024-06-11

**Authors:** Chang Liu, Hao Yu, Hongyi Xia, Ziwei Wang, Bolin Li, Hongmei Xue, Sheng Jin, Lin Xiao, Yuming Wu, Qi Guo

**Affiliations:** 1 Department of Physiology Hebei Medical University Shijiazhuang 050017 China; 2 Department of Reproduction the Second Hospital of Hebei Medical University Shijiazhuang 050017 China; 3 Hebei Collaborative Innovation Center for Cardio-Cerebrovascular Disease Shijiazhuang 050017 China; 4 The Key Laboratory of Neural and Vascular Biology Ministry of Education Shijiazhuang 050017 China; 5 Experimental Center for Teaching Hebei Medical University Shijiazhuang 050017 China; 6 Hebei Key Laboratory of Cardiovascular Homeostasis and Aging Shijiazhuang 050017 China

**Keywords:** heart failure, paraventricular nucleus, sympathetic nerve activity, microglia, inflammation

## Abstract

Sympathetic activation is a hallmark of heart failure and the underlying mechanism remains elusive. Butyrate is generated by gut microbiota and influences numerous physiological and pathological processes in the host. The present study aims to investigate whether the intestinal metabolite butyrate reduces sympathetic activation in rats with heart failure (HF) and the underlying mechanisms involved. Sprague-Dawley rats (220‒250 g) are anaesthetized with isoflurane, and the left anterior descending artery is ligated to model HF. Then, the rats are treated with or without butyrate sodium (NaB, a donor of butyrate, 10 g/L in water) for 8 weeks. Blood pressure and renal sympathetic nerve activity (RSNA) are recorded to assess sympathetic outflow. Cardiac function is improved (mean ejection fraction, 22.6%±4.8% vs 38.3%±5.3%; *P*<0.05), and sympathetic activation is decreased (RSNA, 36.3%±7.9% vs 23.9%±7.6%; *P*<0.05) in HF rats treated with NaB compared with untreated HF rats. The plasma and cerebrospinal fluid levels of norepinephrine are decreased in HF rats treated with NaB. The infusion of N-methyl-D-aspartic acid (NMDA) into the paraventricular nucleus (PVN) of the hypothalamus of HF model rats increases sympathetic nervous activity by upregulating the NMDA receptor. Microglia polarized to the M2 phenotype and inflammation are markedly attenuated in the PVN of HF model rats after NaB administration. In addition, HF model rats treated with NaB exhibit enhanced intestinal barrier function and increased levels of GPR109A, zona occludens-1 and occludin, but decreased levels of lipopolysaccharide-binding protein and zonulin. In conclusion, butyrate attenuates sympathetic activation and improves cardiac function in rats with HF. The improvements in intestinal barrier function, reductions in microglia-mediated inflammation and decreases in NMDA receptor 1 expression in the PVN are all due to the protective effects of NaB.

## Introduction

Chronic heart failure (CHF), the health and economic burden of which has recently been increasing worldwide, remains a leading cause of morbidity and mortality globally [1]. CHF represents an end stage of many cardiovascular diseases (CVDs), including ischaemic heart disease [2], myocardial infarction (MI) [3], hypertension [4], and valvular and vascular heart disease [5]. Despite the availability of classical drugs for CHF treatment, such as angiotensin-converting enzyme inhibitors, beta-adrenergic blockers and angiotensin II receptor blockers, the morbidity and mortality of CHF patients have not greatly improved.

Continual activation of the sympathetic nervous system is strongly related to CHF and has adverse effects on the initiation and development of CHF, especially myocardial remodeling [ 6 – 9]. Thus, inhibiting sympathetic activation is a crucial therapeutic strategy for this complex syndrome. However, the mechanisms underlying sympathetic activation in CHF are not completely understood.

Elevated peripheral sympathetic output in CHF patients is induced by increased activity of specific central neuronal circuits and the release of excitatory neuron transmitters. The paraventricular nucleus (PVN) is located on either side of the superior aspect of the hypothalamus in the third ventricle and plays an important role in autonomic and neuroendocrine functions. Indeed, several studies have shown increased glutamatergic signaling in the PVN in rats with myocardial infarction [10]. In addition, inflammatory mediators can cause central sympathetic excitation [11]. Elevated inflammation in the PVN, which contains presympathetic neurons that project to the rostral ventrolateral medulla, plays an important role in sympathetic excitation in CHF [ 12– 14]. However, the mechanisms underlying the increase in inflammation in the PVN of rodents with CHF are not fully clear.

The gut microbiota, considered an endocrine organ, consists of trillions of bacteria in the gastrointestinal tract and produces hundreds of different metabolites. It is involved in the pathogenesis of a wide range of diseases [15]. The gut microbiota may exert its effects on the brain via various metabolites [16]. Gut microbe fermentation by indigestible dietary fiber results in the production of short-chain fatty acids (SCFAs), including acetate, propionate, and butyrate [17]. The relative abundance of microbes that produce butyrate was found to be reduced in atherosclerosis and heart failure [18]. In addition, butyrate is an important mediator of cardiovascular diseases [19]. However, the central mechanisms of action of butyrate in preventing HF remain unclear, and whether butyrate inhibits sympathetic excitation needs further research.

Accumulating evidence indicates that intestinal barrier dysfunction is an important contributor to the development of CVD. Cardiovascular disease is characterized by increased intestinal barrier permeability [20] related to systemic inflammation induced by leakage of bacterial toxins such as lipopolysaccharide (LPS). Patients with HF exhibit a dysfunctional intestinal barrier and increased intestinal permeability as a result of reductions in ejection fraction (EF) and intestinal blood flow [21], which can lead to increased levels of inflammatory biomarkers [22]. Butyrate and other SCFAs play a role in maintaining the gut mucosal barrier [23] by providing energy for colonocytes. Loss of butyrate-producing bacteria may result in a dysfunctional gut mucosal barrier and trigger inflammation.

In the present study, we hypothesized that gut barrier dysfunction may be involved in sympathetic activation in HF and may be related to microglia-mediated central inflammation associated with gut dysfunction. Using a rat model of left anterior descending artery (LAD) ligation-induced CHF, we explored the effect of butyrate on the gut barrier and microglia-mediated inflammation and its contribution to sympathetic activation.

## Materials and Methods

### Animal experiments

Male Sprague-Dawley (SD) rats weighing 220–250 g were purchased from the Animal Research Center of Hebei Medical University. The animals were housed under controlled environmental conditions on a 12/12-h light/dark cycle at a room temperature of 22±2°C and provided free access to water and standard food. All animal experiment procedures followed the Animal Management Rule of the Ministry of Health, People’s Republic of China (documentation No. 55, 2001) and were approved by the Laboratory Animal Ethical and Welfare Committee of Hebei Medical University.

### Animal model of HF

HF was induced in male SD rats weighing 220–250 g by ligation of the LAD as previously described [24]. The rats were anesthetized with isoflurane (2% in O2; RWD Life Science Co., Shenzhen, China) and incubated for artificial ventilation with a respirator (HX-300; Taimeng Technology Co., Chengdu, China). The rats underwent left thoracotomy to expose the heart. A skin incision (1‒1.5 cm) was made to the left of the breast bone. The pectoralis major and minor muscles were separated by using tissue forceps to expose the fourth intercostal space. Hemostatic forceps were used to bluntly separate the intercostal muscles and the parietal pleura to expose the heart. The LAD was located approximately 2‒3 mm from its origin and ligated. Once the ligation was determined to be successful, the skin incision and chest cavity were closed layer by layer. Sham-operated rats underwent the same surgical procedures except for ligation of the LAD. Isoflurane inhalation was stopped, and the animal was allowed to recover. Penicillin was used to prevent infection, and acetaminophen was used for pain relief to aid recovery.

### Echocardiography

The rats were anesthetized with 2% isoflurane and fixed on an operating table. The rats were subjected to 2D echocardiography with a Vevo 2100 ultrasound device (Fujifilm Visual Sonics, Inc., Toronto, Canada) to determine cardiac function. M-mode echoc ardiography was performed in the parasternal long-axis view and at the level of the left ventricular papillary muscles. The left ventricular end-diastolic diameter (LVDd) and left ventricular end-systolic diameter (LVDs) were measured. Then, the EF, left ventricular end-diastolic volume (LVEDV), and left ventricular end-systolic volume (LVESV) were calculated by using computer algorithms.

### Recording of blood pressure and renal sympathetic nerve activity

Briefly, general anesthesia was induced with 2% isoflurane in 100% O _2_ in rats via inspiration and intubation for artificial ventilation with a respirator. The absence of a withdrawal response to a paw pinch was used to confirm adequate anesthesia. An approximately 2-cm incision was made in the femoral area near the groin, and the right femoral artery was separated from the femoral vein and nerve. A PE 50 tube (SCI, Lake Havasu City, USA) connected to a blood pressure transducer was inserted into the right femoral artery for blood pressure recording. The kidney was exposed in the retroperitoneal position, and the renal nerve around the renal artery was isolated and placed on a pair of platinum electrodes. The nerve and electrode were soaked in paraffin oil to retain nerve activity and decrease noise. The renal sympathetic nerve activity (RSNA) signal was filtered (bandwidth: 30–3000 Hz) and integrated for 0.16 s. The PowerLab 15T data acquisition system was used to monitor blood pressure and RSNA, and the data were saved by using LabChart 7 software (AD Instruments, Sydney, Australia). At the end of the experiment, the maximum RSNA and background electrical noise were obtained by an overdose of sodium pentobarbital (200 mg/kg, i.v.). The electrical noise was subtracted from the integrated RSNA values, and the percentage change in RSNA from baseline was calculated as a percentage of the maximum RSNA. The NMDA-induced change in RSNA from baseline was calculated as a percentage of the basal value.

### PVN microinjection

The rats were anaesthetized with isoflurane (2% in O _2_) and placed in a stereotaxic frame (RWD; Shenzhen, China) in the prone position. According to the rat atlas of Paxinos & Watson (2005), the PVN is located 1.8 mm caudal to the bregma, 0.3 mm lateral to the midline, and 7.8 mm below the skull surface. A glass micropipette attached to a microsyringe via polyethylene tubing was inserted into the PVN and used to deliver N-methyl-D-aspartic acid (NMDA). A total of 200 pmol of 100 nL NMDA was injected into PVN within 5 min. A Powerlab system (AD Instruments) was used to record blood pressure and RSNA simultaneously.

### Masson and hematoxylin-eosin (HE) staining

The animals were deeply anesthetized with isoflurane (2% in O _2_) and the heart and colon tissues were collected, fixed in 4% paraformaldehyde (PFA) and embedded in paraffin. Five-micron-thick sections were cut for HE and Masson’s trichrome staining. Masson’s trichrome staining was used to observe myocardial fibrosis. ImageJ was used to determine the collagen volume fraction (CVF; CVF=collagen area in the myocardial interstitium/total field area). Histopathological images were obtained and analyzed under a digital slide scanner (Pannoramic MIDI; 3D HISTECH, Budapest, Hungary).

### Western blot analysis

The animals were deeply anesthetized with isoflurane (2% in O _2_). Fresh brain tissue containing the PVN and colonic tissues was collected and rinsed with saline at 4°C, and brain slices (250 μm) were cut with a vibratome (VT1200S; Leica, Wetzlar, Germany). The tissue was placed in an ultrasound machine for lysis with lysis buffer (SW104-02; SEVEN, Beijing, China), after which the protein supernatants were collected for analysis. Equal amounts of protein were separated via SDS-PAGE (10% separating gel and 4% stacking gel; 80 V; 150 min) and subsequently transferred to PVDF membranes (Millipore, Billerica, USA), which were blocked with 0.1% Tween-20 in Tris-buffered saline (TBST) containing 5% skim milk for 1.5 h at room temperature. The membranes were then incubated with primary antibodies against NOD-like receptor protein 3 (NLRP3, 1:1000, 19771-1-AP; Proteintech, Wuhan, China), N-methyl-D-aspartic acid receptor 1 (NMDAR1, 1:1000; ab109182; Abcam, Cambridge, USA), interleukin 10 (IL-10, 1/1000, 60269-1-Ig; Proteintech), GPR109A (1:1000, NBP1-92180; Novus, Shanghai, China), occludin (1:5000, 27260-1-AP; Proteintech), and zona occludens1 (ZO-1, 1:5000, 21773-1-AP; Proteintech) overnight at 4°C and then with HRP-conjugated goat anti-rabbit IgG (SA00001-2; Proteintech) or goat anti-mouse IgG (SA00001-1-A; Proteintech) secondary antibodies for 1.5 h at room temperature. The protein bands on the PVDF membrane were detected by using a chemiluminescent substrate system (ChemiScope 6200; Clinx Science Instruments Co., Ltd., Shanghai, China). The protein concentration was normalized to that of GAPDH.

### Measurement of norepinephrine (NE), atrial natriuretic peptide (ANP), brain natriuretic peptide (BNP) and lipopolysaccharide binding protein (LBP) concentrations

Eight weeks after the animal model was established, the rats were anesthetized for blood collection from the inferior vena cava and cerebrospinal fluid (CSF). ELISA kits (COIBO BIO, Shanghai, China) were used to measure the levels of NE, ANP, BNP and LBP according to the manufacturer’s instructions. The absorbance was read at a wavelength of 450 nm with a microplate reader (PowerwaveXS2; BioTek, Winooski, USA).

### Immunofluorescence staining

After anesthetization with sodium pentobarbital (50 mg/kg, i.p.), the rats were sequentially perfused with saline (0.9% NaCl) and 4% paraformaldehyde transcardially. Brain tissues containing the PVN were collected, fixed in 4% PFA a week and subsequently incubated in 30% sucrose in PBS twice until they sank to the bottom. Coronal sections were cut at 25 μm on a cryostat (CM1950; Leica). For double immunohistochemical staining, PBS was used to wash the brain sections three times, after which the sections were incubated overnight with primary antibodies, including anti-rabbit CD86 (13395-1-AP; Proteintech), anti-rabbit CD206 (ab64693; Abcam), anti-mouse ionized calcium binding adapter molecule 1 (Iba-1, GT10312; GeneTex, Irvine, USA) in 0.25% Triton X-100 in PBS (PBS-T) at 4°C for 12 h. The brain sections were subsequently washed three times with PBS before incubation with fluorescein-conjugated goat anti-rabbit IgG (ab150083; Abcam) or goat anti-mouse IgG (SA00013-1; Proteintech, Wuhan, China) secondary antibody for 2 h at room temperature. The tissues were fixed on slides, dried, dehydrated and covered. Fluorescence images were acquired with a fluorescence microscope (DM6 B Thunder imager; Leica). Five fields (20×) on each slide were randomly selected for analysis; the average value was used for analysis.

### Statistical analysis

Data are expressed as the mean±SD. SPSS 20.0 software (IBM SPSS, Armonk, USA) was used for analysis. One-way ANOVA was used to compare the differences among groups. *P*<0.05 was considered to indicate statistical significance.

## Results

### NaB enhances cardiac function and attenuates cardiac remodeling in HF rats

**Figure 1:**
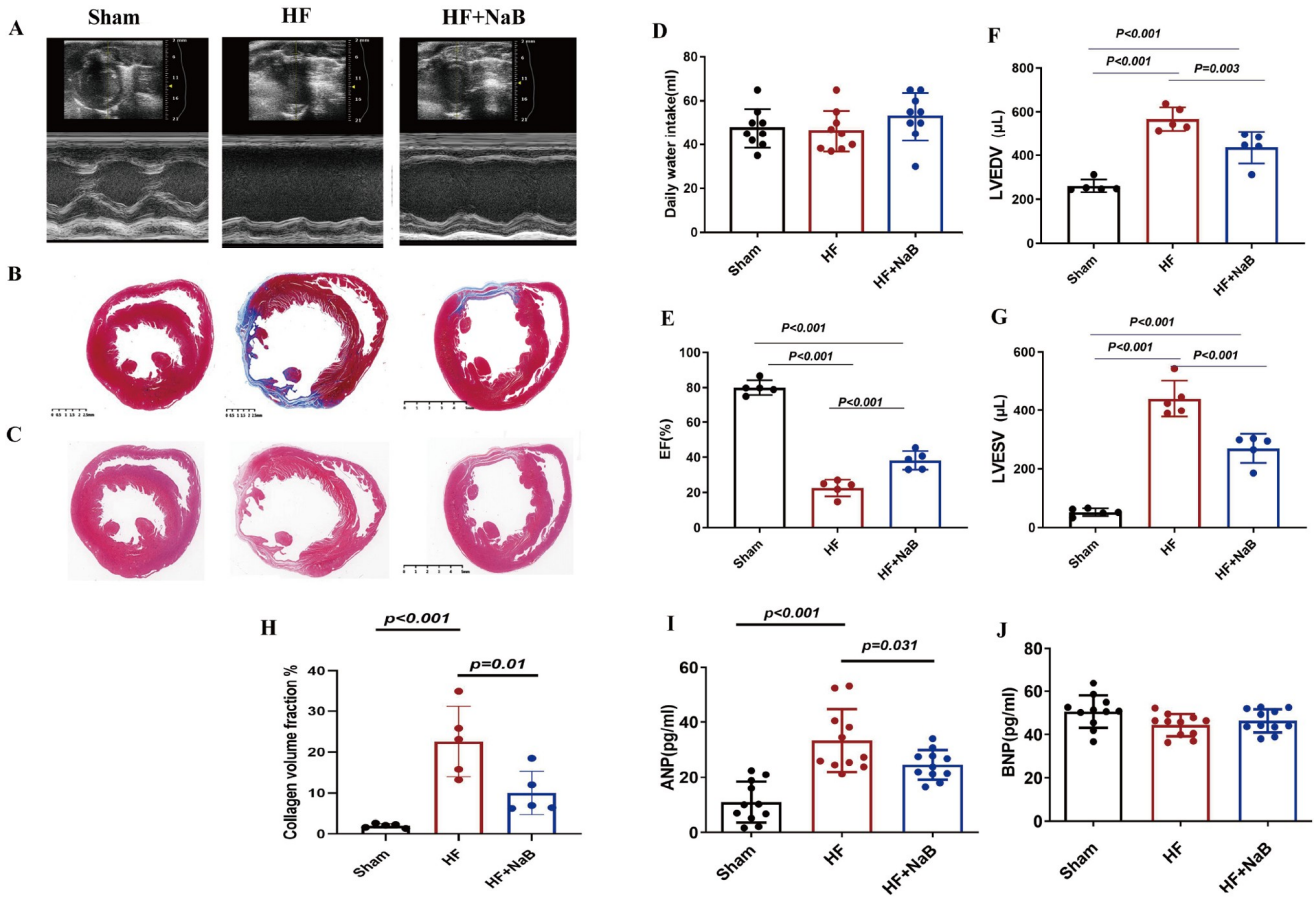
Figure 1 NaB protects against cardiac dysfunction and remodeling in HF rats (A) Echocardiographic tracings, (B) Masson staining, (C) HE staining, (D) daily water intake, (E) ejection fraction (EF), (F) left ventricular end-diastolic volume (LVEDV), (G) left ventricular end-systolic volume (LVESV), (H) collagen volume fraction, (I) ANP, and (J) BNP in rats with heart failure (HF), NaB-treated HF model rats and sham rats. Data are presented as the mean±SD; n=5‒10. One-way ANOVA was used.

To determine whether NaB improves cardiac function and remodeling, echocardiography and morphological staining were used to test animals in different groups. Figure 1A shows the EF, LVEDV and LVESV of the sham-operated, HF and HF+NaB groups at 6 weeks after surgery according to echocardiography. Compared with sham rats, HF model rats had a significantly lower mean EF (80.0%±4.2% vs 22.6%±4.8%, *P* <0.001; Figure 1E) but greater LVEDV (260.9±28.8 μL vs 567.2±53.7 μL, *P*<0.001; Figure 1F) and LVESV (50.3±12.8 μL vs 442.5±59.6 μL, *P*<0.001; Figure 1G). However, HF model rats treated with NaB exhibited improved cardiac function (mean EF, 22.6%±4.8% vs 38.3%±5.3%, *P*<0.001; LVEDV, 567.2±53.7 μL vs 436.0±73.3 μL, *P*<0.001; and LVESV, 442.5±59.6 μL vs 268.6±49.0 μL, *P*<0.001; Figure 1E‒G). Therefore, NaB could effectively attenuate the deterioration of cardiac function in HF model rats.

Heart tissue was subjected to HE and Masson’s trichrome staining after treatment with NaB for 8 weeks ( Figure 1B,C). Histological examination of cardiac tissues further confirmed that myocardial fibrosis was reduced in NaB-treated HF model rats compared with untreated HF model rats (CVF, 22.6%±8.6% vs 10.0%±5.3%, *P*=0.010; Figure 1H). Furthermore, ANP and BNP were measured to evaluate cardiac tissue damage ( Figure 1I,J). In particular, the results revealed a remarkable increase in ANP in the HF group compared with the sham group (ANP, 11.0±7.4 ng/mL vs 33.2±11.4 ng/mL, *P*<0.001), and this increase was reversed by NaB administration (ANP, 33.2±11.4 ng/mL vs 24.5±5.4 ng/mL, *P*=0.031). However, there was no significant difference in BNP.

### The decrease of sympathetic response to NMDA is associated with NaB administration in HF rats

**Figure 2:**
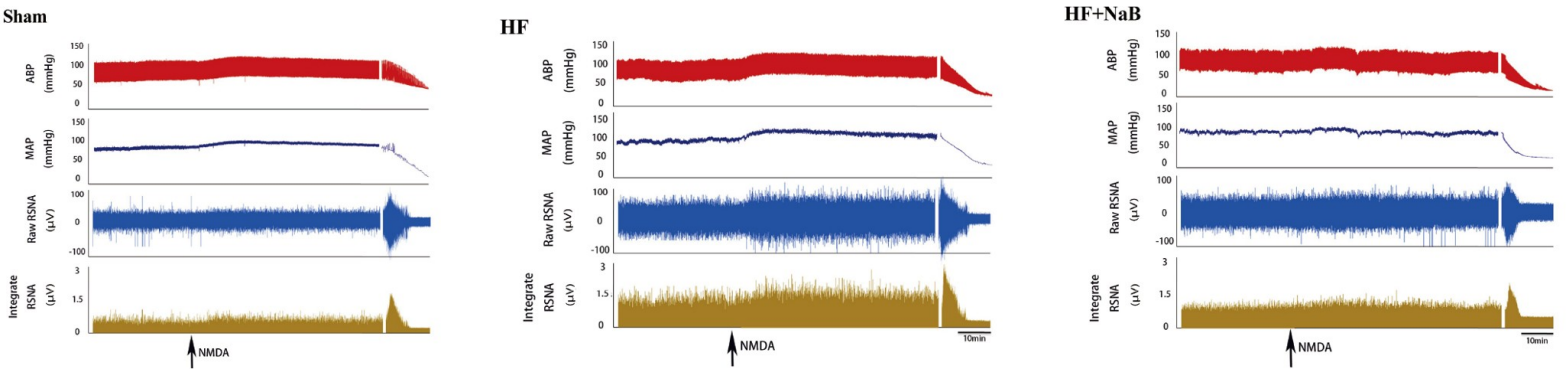
Figure 2 Effect of NaB on basal renal sympathetic nerve activity (RSNA), arterial blood pressure (ABP) and NMDA-induced changes in RSNA and ABP in HF model rats Raw traces showing RSNA after microinjection of NMDA (200 pmol, 100 nL) into the paraventricular nucleus of the hypothalamus. “↑” indicates microinjection of NMDA.

**Figure 3:**
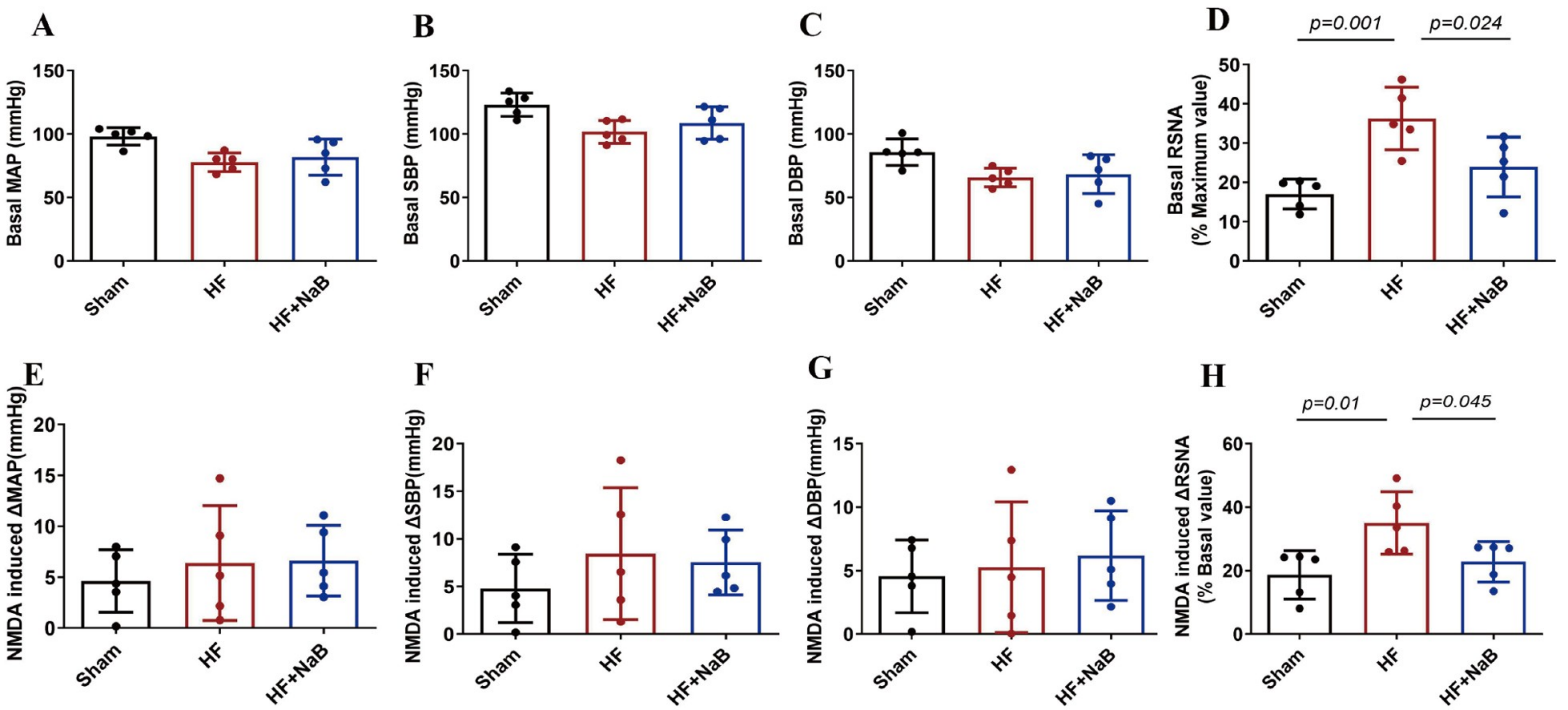
Figure 3 Summary data showing that NaB decreases sympathetic outflow in HF model rats (A‒D) Basal mean arterial pressure (MAP), systolic blood pressure (SBP), diastolic blood pressure (DBP) and RSNA. (E‒H) NMDA-induced changes in MAP, SBP, DBP, and RSNA. Data are presented as the mean±SD; n=5. One-way ANOVA was used.

To clarify the role of NaB in sympathetic nerve activity in HF rats, we assessed its effect on basal and NMDA-induced sympathetic outflow by recording RSNA and BP. Figures 2 and 3 show the original traces and summary data of the sympathetic responses to the microinjection of NMDA into the PVN of HF model rats treated with or without NaB and sham rats. Compared with the sham rats, the HF model rats exhibited increased mean basal RSNA (17.1%±3.8% vs 36.3%±7.9% Max, *P*=0.001), and NaB reduced the mean basal RSNA (36.3%±7.9% vs 23.9%±7.6% Max, *P*=0.024). Microinjection of NMDA into the PVN significantly increased the mean RSNA in HF model rats compared with sham rats (35.6%±9.5% vs 18.7%±7.6%, *P*=0.010). The mean RSNA in response to NMDA was significantly decreased after NaB treatment (35.6%±9.5% vs 22.8%±6.4%; *P*=0.045). However, blood pressure fluctuations were not different between the groups.

### NaB suppresses NE levels in HF rats

**Figure 4:**
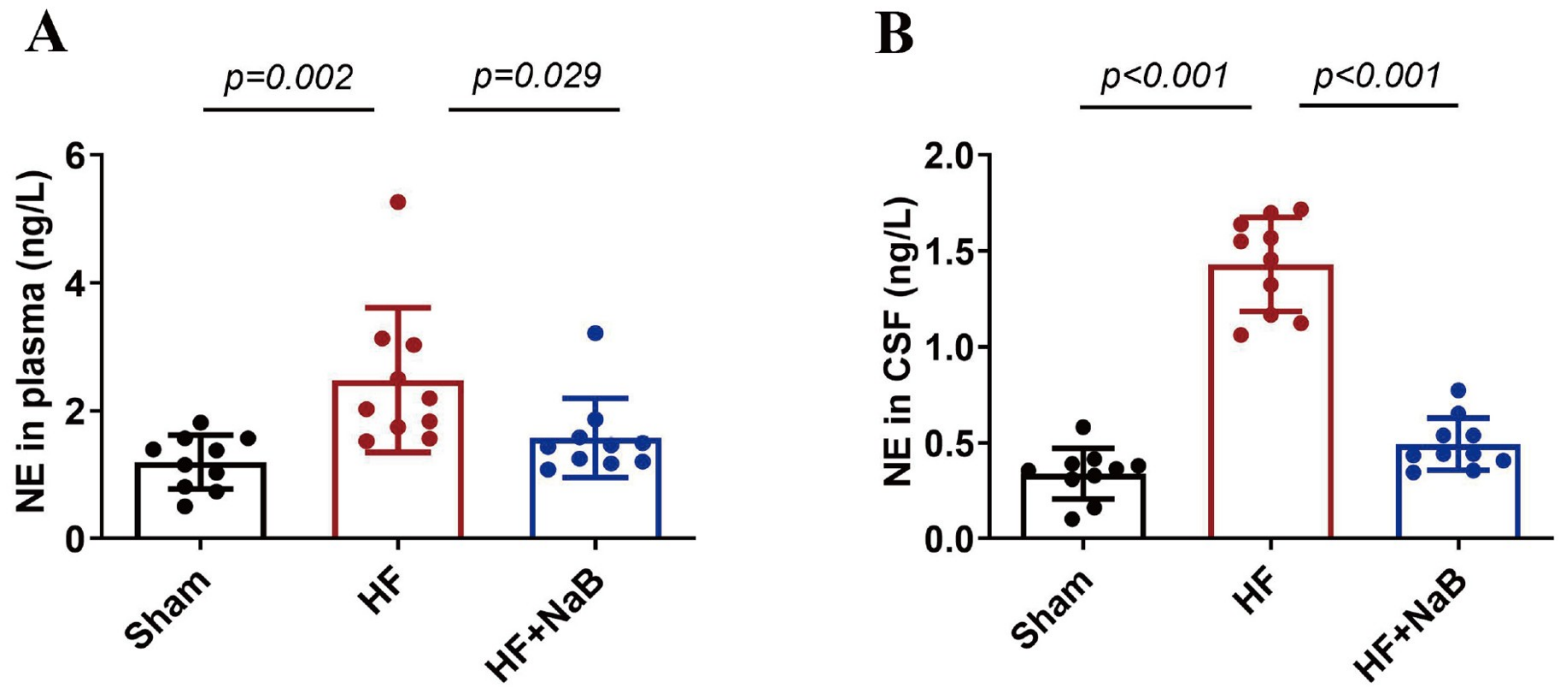
Figure 4 NaB reduces the levels of norepinephrine (NE) in HF rats NE levels in plasma (A) and cerebrospinal fluid (B) in rats. Data are presented as the mean±SD; n=10. One-way ANOVA was used.

Compared with sham rats, HF model rats showed significantly increased mean plasma and CSF NE levels (plasma, 1.2±0.4 ng/L vs 2.5±1.1 ng/L, *P*=0.002; CSF, 0.3±0.1 ng/L vs 1.4±0.3 ng/L, *P*<0.001) ( Figure 4). However, treatment with NaB significantly decreased the mean NE concentration (plasma, 2.5±1.1 ng/L vs 1.6±0.6 ng/L, *P*=0.029; CSF, 1.4±0.3 ng/L vs 0.5±0.1 ng/L, *P*<0.001).

### NaB alters the polarization of microglia in the PVN of HF model rats

**Figure 5:**
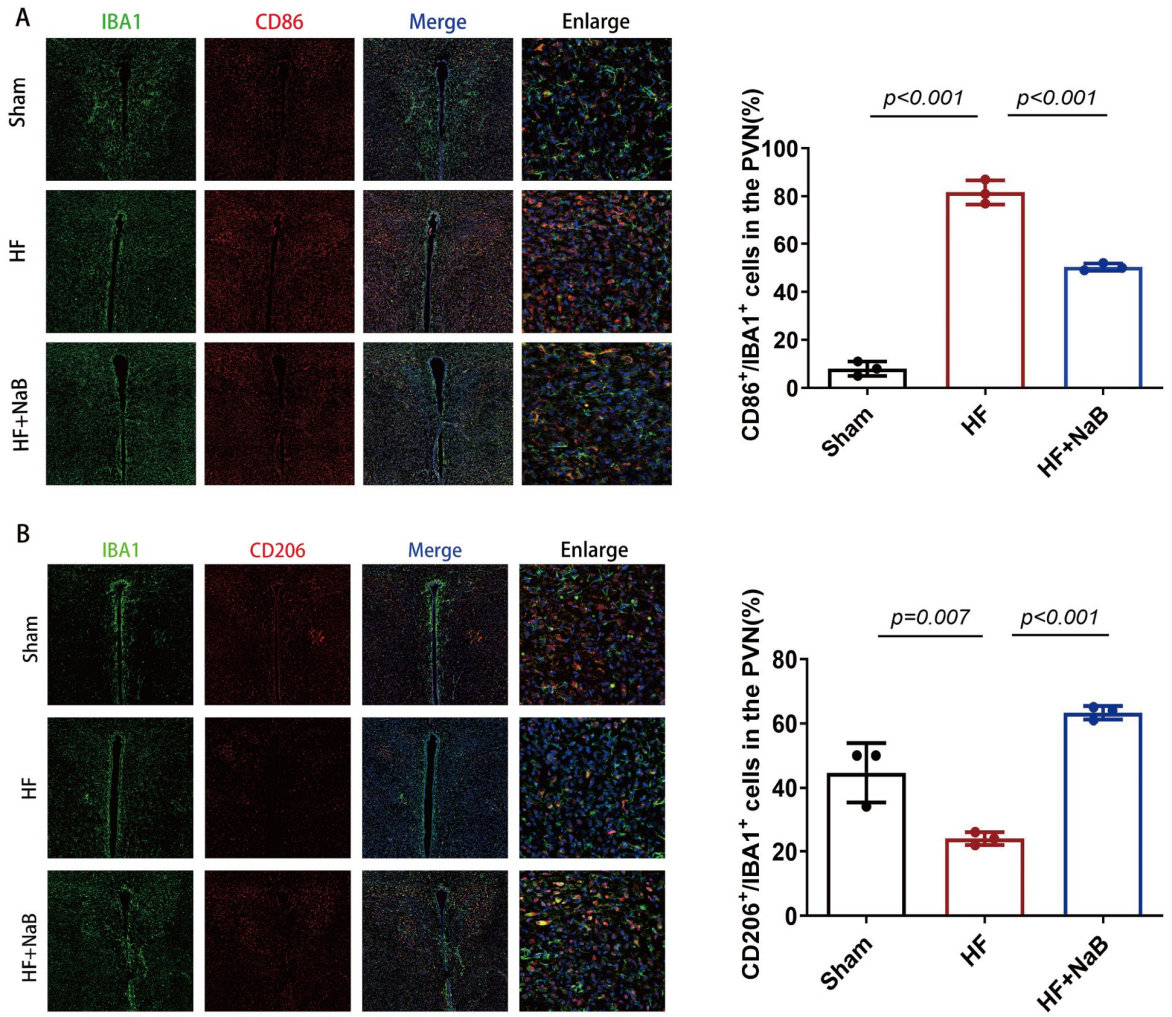
Figure 5 Polarization of microglia in the PVN of HF model rats treated with NaB (A,B) Immunofluorescence staining of CD86+cells (red) and CD206+ cells (red) in Iba1 (green) in the PVN of rats. Data are presented as the mean±SD of the proportions of CD86+/Iba1+ and CD206+/Iba1+ cells in the PVN; five consecutive visual fields on one slide and two slides per rat were analyzed (n=3). One-way ANOVA was used.

Immunofluorescence staining was used to evaluate the polarization of microglia ( Figure 5). In the PVN, the number of CD86-positive microglia was significantly greater in HF model rats than in sham rats, with a significant decrease in the number of CD206-positive microglia. In contrast, HF model rats treated with NaB presented an increased proportion of M2 microglia and a decreased proportion of M1 microglia in the PVN.

### NaB inhibits neuroinflammation and decreases NMDAR1 protein level in PVN of HF rats

**Figure 6:**
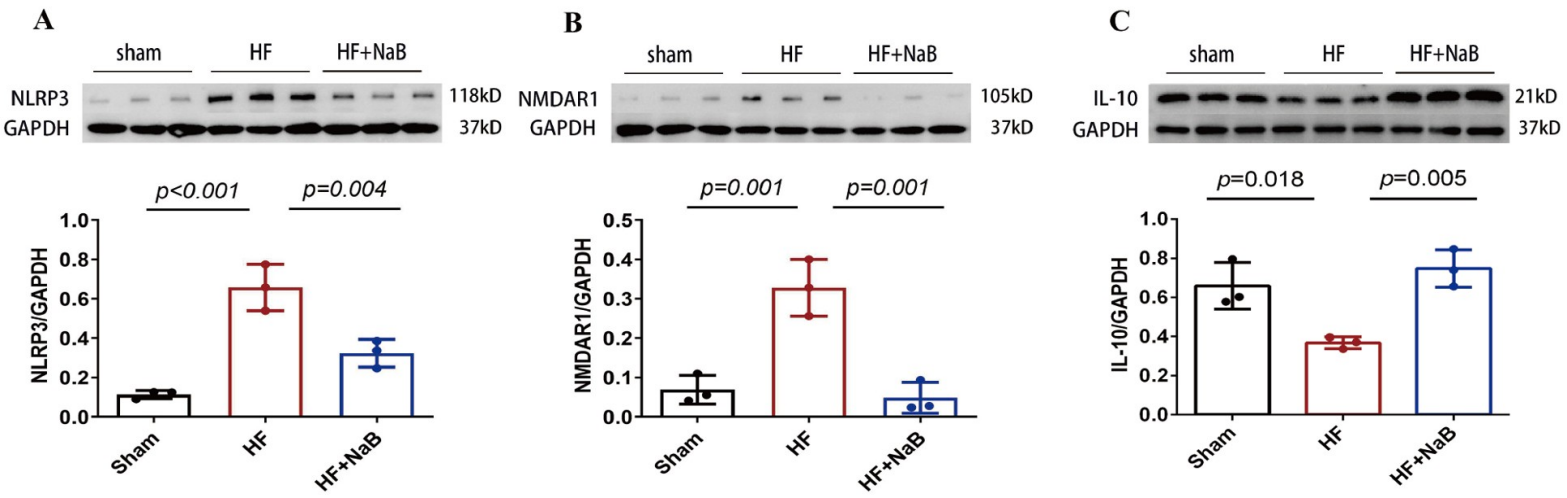
Figure 6 NaB downregulates inflammation-related protein and NMDAR1 level in the PVN Protein levels of (A) NLRP3, (B) NMDAR1, and (C) IL-10 in the PVN of rats. Data are presented as the mean±SD; n=3. GAPDH was used for normalization. One-way ANOVA was used.

The observation that NaB could affect sympathetic activation in HF rats prompted us to dissect the underlying molecular mechanisms. We conducted western blot analysis to investigate the inflammation-related proteins and the expression of NMDA receptor in PVN. Our results showed that the protein levels of NLRP3 and NMDAR1 were significantly increased in HF model rats, while the level of IL-10 was decreased ( Figure 6). However, compared with HF, the exogenous administration of NaB for 8 weeks exhibited a significantly decrease in NLRP3 and NMDAR1, and an increase in IL-10.

### NaB protects intestinal mucosal barrier function in HF model rats

**Figure 7:**
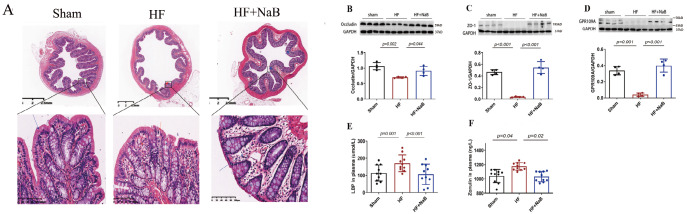
Figure 7 Effect of NaB on intestinal mucosal barrier function (A) HE staining of the colon. Protein levels of (B) occludin, (C) ZO-1, and (D) GPR109A in the colon of rats; n=4. GAPDH was used for normalization. Plasma levels of (E) LBP and (F) zonulin in different groups; n=10. Data are presented as the mean±SD. One-way ANOVA was used.

Histological examination of colonic tissue revealed that NaB reversed intestinal tissue injury in HF model rats ( Figure 7A). The protein levels of occludin, ZO-1, and GPR109A were significantly decreased in the intestinal tissue of HF model rats, and these changes were reversed by treatment with NaB (GPR109A, *P*<0.001; occludin, *P*=0.044; ZO-1, *P*<0.001) ( Figure 7B‒D). We further assessed the permeability of intestinal mucosal barrier and found that compared with sham rats, HF model rats presented increased mean plasma levels of LBP (114.0±47.0 μmoL vs 170.6±48.5 μmoL, *P*=0.04) and zonulin (1040.5±91.3 mg/L vs 1176.0±53.8 mg/L, *P*=0.001). Treatment with NaB for 8 weeks decreased the mean levels of LBP (170.6±48.5 μmoL vs 106.8±59.3 μmoL, *P*=0.02) and zonulin (1176.0±53.8 mg/L vs 1032.0±70.5 mg/L, *P*<0.001).

## Discussion

In the present study, cardiac function was decreased in rats with HF, while sympathetic outflow and NE levels were increased. Acute microinjection of NMDA into the PVN induced a greater increase in RSNA in HF model rats than in sham rats. The proportion of M1 microglia in the PVN was greater in HF model rats than in sham rats. Inflammation in the PVN was increased, and intestinal mucosal barrier function was impaired in HF model rats. Treatment with butyrate significantly improved intestinal mucosal barrier function and decreased inflammation to protect against sympathetic activation in HF rats.

Heart failure is a multifactorial, life-threatening syndrome characterized by high morbidity and mortality. Among the multiple potential etiologies, ischaemic cardiomyopathy is a major risk factor for heart failure [25], accounting for up to 26.5% of heart failure cases worldwide [ 26 – 28]. However, 20%–30% of patients who experience MI develop post-MI HF within one year after percutaneous coronary intervention [29]. Based on accumulating evidence, the central nervous system, especially the sympathetic nervous system, strongly affects HF pathophysiology [ 30, 6]. Renal sympathetic nerve activity is a common measure for assessing sympathetic activity. NE is a neurotransmitter released from sympathetic nerve endings, and its level indicates sympathetic outflow [ 31, 32]. In the present study, in HF model rats, basal RSNA was increased, and this change was accompanied by an increase in the plasma and CSF NE concentrations. Consistent with the findings of previous studies, sympathetic excitation is a key phenomenon in HF and is closely related to heart remodeling and tissue damage [33]. MI-induced HF is associated with cardiac injury, including cardiac dysfunction and tissue damage. Therefore, in our study, cardiac function was decreased and myocardial fibrosis was increased in HF rats. ANP and BNP, which are encoded by nppa and nppb, respectively, are considered cardiac hypertrophy-related genes [ 34, 35]. ANP and BNP elevation is commonly found in heart failure patients and is used as an index for the diagnosis of heart failure. We found that plasma level of ANP was greater. In contrast, BNP was normal in the MI-induced HF model, which may be an adaptive phenomenon after ventricular remodeling.

The PVN contains different types of neurotransmitters that control various physiological functions [36]. Previous studies have shown that NMDAR, an ionotropic glutamate receptor [37], is expressed in PVN neurons and is involved in the mediation of sympathetic activity in hypertension [ 38, 39]. NMDARs are macromolecular structures formed by different combinations of the subunits NMDAR1, NMDAR2 and NMDAR3 [40]. However, NMDAR1 expression is significantly elevated in the PVN of rats with HF [41]. NMDAR blockade in the PVN with the NMDAR antagonist D(-)-2-amino-5-phosphonopentanoic acid (AP5) significantly reduces sympathetic outflow and arterial blood pressure in hypertensive rats [42], but microinjection of AP5 into the PVN does not significantly alter MAP or sympathetic nerve activity in healthy rats [43]. In our study, microinjection of NMDA into the PVN elevated RSNA in HF model rats and increased the response to NMDA. Additionally, the level of NMDAR1 was increased in the PVN. Hence, excessive sympathetic outflow may be attributed to the upregulation of NMDARs in the PVN in HF model rats.

Microglia play a vital role in maintaining brain homeostasis, particularly by regulating neuroinflammation [ 44, 45]. CD86 is a marker of proinflammatory M1 microglia, and anti-inflammatory M2 microglia expresses CD206 [ 46, 47]. Neuroinflammation is strongly involved in the activation of sympathetic nerves [48]. Microinjection of IL-17A into the lateral ventricle or the PVN induces an excitatory response involving increases in blood pressure, heart rate and RSNA [49]. Suppressing microglial M1 polarization and neuroinflammation in the rostral ventrolateral medulla ameliorates sympathetic hyperactivity in stress-induced hypertensive rats [50]. We found an increased proportion of M1 microglia in the PVN of rats with HF, which indicates that microglia are polarized toward the M1 phenotype in HF model rats. In addition, the levels of inflammatory markers, such as NLRP3, were significantly increased, while the level of IL-10 was notably decreased. Thus, microglia-mediated neuroinflammation contributes to sympathetic activation in rats with HF. A previous study demonstrated that IL-1β and tumor necrosis factor-α, which are separately released by activated glial cells, amplify NMDAR-mediated synaptic currents [ 51, 52]. Collectively, our findings also provide evidence that microglia-mediated neuroinflammation is related to increased sympathetic excitability via NMDARs.

Additionally, HF model rats exhibited impairment of the intestinal mucosal barrier. The intestinal tract is the central site for the exchange of substances between the internal and external environments, and increasing evidence has suggested that neurotransmitters, immune signals and neuropeptides produced in the gut may interact with the brain [ 53, 54]. It is possible that intestinal bacteria metabolize arachidonic acid and LPS, which leak into the circulatory system and are triggered by gut dysbiosis [55]. Moreover, these metabolites can stimulate microglial activation and provoke neuroinflammation [ 56– 58]. We found decreased levels of occludin and ZO-1 in the colon of HF model rats; these proteins are tight junction proteins in the colon and are fundamental for maintaining the integrity of the epithelial barrier [ 59 , 60]. Additionally, the plasma levels of LBP and zonulin were increased in HF model rats. Thus, injury to the gut increases the entry of LPS into the circulation, which activates microglia, thus leading to sympathetic activation.

There is a strong relationship between the gut microbiome and neurological disorders [61]. Butyrate, an SCFA, has an anti-inflammatory effect on microglia and blocks abnormal phagocytosis of microglia [ 62, 63]. Furthermore, GPR109A is a receptor for butyrate in the colon that is encoded by *Niacr1* [64]. In our study, NaB effectively improved intestinal barrier function and reduced LPS levels in the plasma of HF model rats, in addition to inhibiting microglia-mediated inflammation in the PVN. Consistent with what was observed in previous studies, NaB protected against pathological damage to cardiac tissue in HF model rats in this study [65]. Thus, the beneficial effect of NaB could be attributed to the inhibition of inflammation-mediated sympathetic activation.

In summary, the present study revealed that the intestinal metabolite butyrate reduced sympathetic hyperactivation and improved cardiac function in rats with chronic HF. The ameliorative effect of butyrate may be related to improved intestinal barrier function and reduced microglia-mediated inflammation in the PVN. We provide a new experimental basis and target for managing HF. However, there are several limitations to this study. We did not provide direct evidence that sympathetic activation leads to gut dysfunction in HF or is the target of NaB. We will continue to study this topic further in our future studies.
